# Geographical inequalities in uptake of NHS funded eye examinations: Poisson modelling of small-area data for Essex, UK

**DOI:** 10.1093/pubmed/fdx058

**Published:** 2017-06-17

**Authors:** Darren Shickle, Tracey M Farragher, Chris J Davey, Sarah V Slade, James Syrett

**Affiliations:** 1 Academic Unit of Public Health, Leeds Institute of Health Sciences, University of Leeds, Leeds, UK; 2 Bradford School of Optometry and Vision Science, University of Bradford, Bradford, UK; 3 evolutio Care Innovations Ltd, Henley-on-Thames, UK

**Keywords:** epidemiology, eye disorders, socioeconomics factors

## Abstract

**Background:**

Small-area analysis of National Health Service (NHS)-funded sight test uptake in Leeds showed significant inequalities in access among people aged <16 or ≥60.

**Methods:**

Data were extracted from 604 126 valid General Ophthalmic Services (GOS)1 claim forms for eye examinations for Essex residents between October 2013 and July 2015. Expected GOS1 uptake for each lower super output area was based on England annual uptake. Poisson regression modelling explored associations in GOS1 uptake ratio with deprivation.

**Results:**

People aged ≥60 or <16 living in the least deprived quintile were 15% and 26%, respectively, more likely to have an NHS funded eye examination than the most deprived quintile, although all are equally entitled. GOS1 uptake is higher in the more deprived quintiles among 16–59-year old, as means tested social benefits are the main eligibility criteria in this age-group. Inequalities were also observed at local authority level.

**Conclusions:**

Inequalities in access among people ≥60 years were not as large as those reported in Leeds, although inequalities in <16-year old were similar. However, demonstrable inequalities in this data set over a longer time period and a larger and more diverse area than Leeds, reinforce the argument that interventions are needed to address eye examination uptake inequalities.

## Introduction

Preventable sight loss is an indicator within the Public Health Outcomes Framework for England, 2016–19.^1^ The indicator is defined in terms of the incidence of Certificates of Vision Impairment (CVI) issued for glaucoma, diabetic eye diseases, age-related macular degeneration (AMD) and for all causes.

Socio-economic deprivation in the UK has also been associated with presentation with more advanced field loss due to glaucoma;^2–6^ lower uptake of diabetic retinopathy screening and higher prevalence of sight-threatening retinopathy;^7–11^ and later presentation with AMD.^12^

Malik *et al.*^13^ examined variation in the standardized rates of CVI submitted for all Primary Care Trusts (PCT) in England. There was an 11-fold variation in CVI registration rates, but only a very weak association with deprivation. The authors suggested that most of the variation may be due to the quality of data with under-reporting by clinicians. While this may be true, PCT boundaries may have been too large a geographical unit for analysis, as there will be considerable variation in deprivation within PCT areas.

To better understand socio-demographic inequalities in sight loss that was potentially preventable, it is necessary to explore variation in the uptake of eye examinations within primary care where most cases are initially detected. In the UK, these are usually conducted within primary optometric practice, either funded privately or via the National Health Service (NHS). In England, the NHS funds eye examinations under a General Ophthalmic Services (GOS) contract with optometrists, for all children aged under 16 and children aged 16, 17 or 18 who are in full time education, people aged 60 and over, people on specified means tested benefits (e.g. Income Support, Income-based Jobseeker's Allowance, Pension Credit Guarantee Credit, income-based Employment and Support Allowance), and those suffering from or pre-disposed to eye disease.^14^ The last time official data were published^15^ in 2005–6, 68.6% of all sight tests conducted in England were funded by the NHS. The Optical Confederation estimated that 71.1% of sight tests performed in the UK in 2013–14 were funded by the NHS (although this included data for Scotland where NHS funded sight tests are available to all Scottish residents).^16^

We have previously conducted a small-area analysis of 17 680 GOS1 claim forms for eye examinations conducted in Leeds during a 5-week census period in 2011.^17^ People aged 60 or over living in the least deprived quintile were 71% more likely to have a NHS funded eye examination than someone in that age-group in the most deprived quintile. People aged 16 years or under living in the least deprived quintile were 23% more likely to have an NHS funded eye examination compared with the most deprived quintile. All people aged 60 and over and under 16 are equally entitled to an NHS funded eye examination. GOS1 uptake was higher in the more deprived quintiles among 16–59-year old, as means tested social benefits were the main eligibility criteria in this age-group.

In 2014–15,^18^ Essex was the only area of England to report General Ophthalmic Services Activity Statistics based on nearly all (94.4%) of GOS1 claims, as forms are processed electronically. All paper GOS claim forms in Essex are scanned with optical character recognition software, checked by hand and then algorithm validated. The published routine data from the remaining 24 areas of England were extrapolated from a small sample of GOS claim forms (ranging from 1.6 to 51.6%, with a skew towards smaller samples of <2%) and the sub-totals were grossed up accordingly.^18^ NHS Digital who publish the data do not explain how the GOS1 sampling frames in the areas were constructed. Due to a lack of electronic data, the small-area analysis in Leeds^17^ required a manual data entry of paper GOS1 claim forms. Essex, therefore, offered an opportunity to utilize a significantly larger data set over a longer period to analyse inequalities of access than would be possible from a manual data entry.

## Methods

### Setting

Essex is a county in South East England. The county comprises 12 local authority areas. Compared to England, Essex local authorities tend to serve populations with: higher percentages of people over the age of 65; lower long-term unemployment rates; lower proportions of the population from ethnic minorities; similar percentages of people reporting limiting long-term illness or disability; and, lower all cause standardized mortality ratios.^19^

### Cohort

Data were obtained for Essex from the provider which manages the processing of GOS1 claim forms on behalf of the Essex Area Team of NHS England. Data from all GOS1 application forms for an NHS funded sight test within Essex between October 2013 and July 2015 were entered onto a database (*n* = 855 079). The initial data set contained accepted GOS1 claims and rejected claims that were returned to the GOS contractor for correction and resubmission. Only accepted claims were included (*n* = 795 940, 6.9% removed) within subsequent analysis to prevent double counting of resubmitted GOS1 forms. Data were also removed if the patient resided outside of the Essex boundaries but had travelled into the region to have an NHS funded sight test (171 020 (20.0%) removed). A further 20 584 tests (2.4%) were excluded as it was not possible to identify a legitimate postcode for the patient and 628 (0.07%) tests were removed as age at time of the test was not recorded; leaving 603 508 tests.

### Statistical analysis

The small-area analysis was based on lower super output areas (LSOAs) that are built from groups of neighbouring 2011 census output areas (OAs).^20^ LSOAs are automatically generated to be as consistent as possible in population size (1000–3000) and characteristics of the component communities in each LSOA.

The outcome calculated was the ratio between the ‘actual uptake’ of GOS1 within a LSOA and the ‘expected GOS1 uptake’ within that LSOA. The ‘actual GOS1 uptake’ was calculated by using the postcode of residence, detailed on each GOS1 record, mapped to LSOA, so providing the aggregated number of GOS1 claims within each LSOA. The number of claims within each LSOA were aggregated based on the age groups for age at test (<16, 16–59 and 60 over).

The ‘expected number of GOS1 uptake’ for each LSOA was based on the 2014–15 GOS1 national annual uptake rate^18^ applied to mid-year 2013 population estimates^21^ for each LSOA. The expected rates were estimated for each of the three age groups by collating the national GOS1 uptake by reasons for uptake i.e. age-group under 16 (GOS1 eligibility reason children 0–15), age-group 60 and over (GOS1 eligibility reason 60 and over) and age-group 16–59 (all other GOS1 eligibility reasons).

A GOS1 uptake ratio for a LSOA greater than one means that the GOS1 uptake in that area is above that expected nationally while a GOS1 uptake ratio for a LSOA less than one equates to GOS1 uptake that is lower than expected nationally in that area.

The geographical variation of the GOS1 uptake ratio and the deprivation indices across the LSOA areas in Essex are shown via maps. The boundary files for the LSOAs within Essex were provided by the Digimap service (^©^Crown Copyright/database right 2016. An Ordnance Survey/EDINA supplied service). Deprivation was measured by the Indices of Multiple Deprivation ranking as at 2010 for each LSOA, with the rankings in the Essex localities converted to quintiles.^22^

Unadjusted GOS1 uptake ratio by Index of Multiple deprivation (IMD) quintile was calculated to initially explore the relationship between GOS1 uptake and deprivation. Poisson regression models were used to explore this association further and provide estimates for GOS1 uptake ratio ratios for each deprivation quintile. In these models, the crude GOS1 uptake ratio is included as two separate components: the actual number of GOS1 uptakes per LSOA is the dependent variable while the expected number of GOS1 uptakes per LSOA is included as the exposure variable.

Separate Poisson models were undertaken for each of the three age groups to explore if the associations with deprivation differed by age/eligibility criteria. As each of the three age groups have different reasons for GOS1 uptake—all under 16 and 60 and over are eligible while those using GOS1 between 16 and 59 are eligible for various reasons related to receiving benefits and those suffering from or pre-disposed to eye disease, then the association between GOS1 uptake ratio and deprivation is expected to be very different in these three age groups i.e. age-group is an effect modifier rather than a potential confounder.

To explore whether differences between local authorities accounted for any association between deprivation and the GOS1 uptake rate ratios (URRs), the models were firstly adjusted by the 12 local authority areas in Essex. To then show whether the associations with deprivation differed in the local authority areas, interaction terms between local authority and deprivation quintile were added to the models.

Differences in the GOS1 URRs are also shown and estimated from these models to ease interpretation in exploring the differences between deprivation quintiles and local councils rather than directly comparing the URRs. The comparators were selected as the first groups in the variable i.e. the most deprived quintile and Basildon Local Council (first alphabetically).

No personal identifiable data were used and hence ethics approval was not required. There was a data sharing agreement between NHS England, evolutio Care Innovations Ltd., The College of Optometrists and University of Leeds.

## Results

**Fig. 1:**
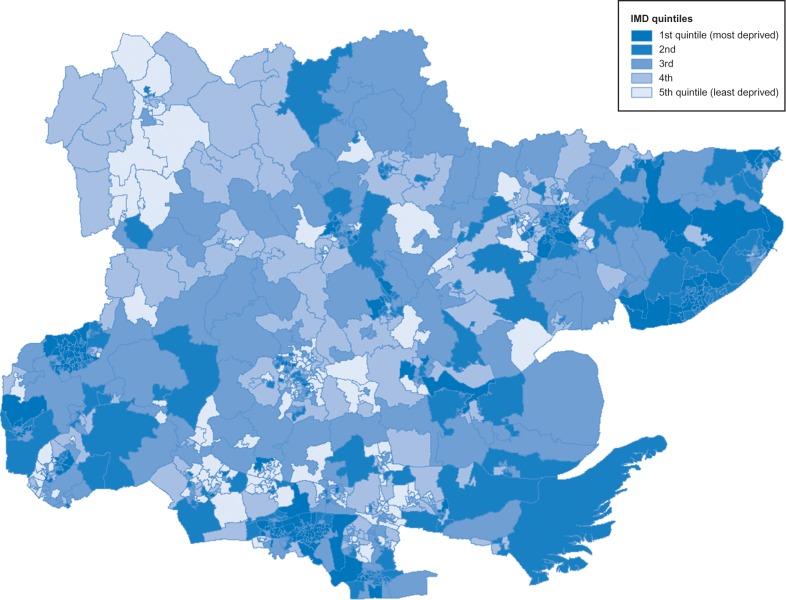
LSOAs by IMD quintiles.

**Fig. 2:**
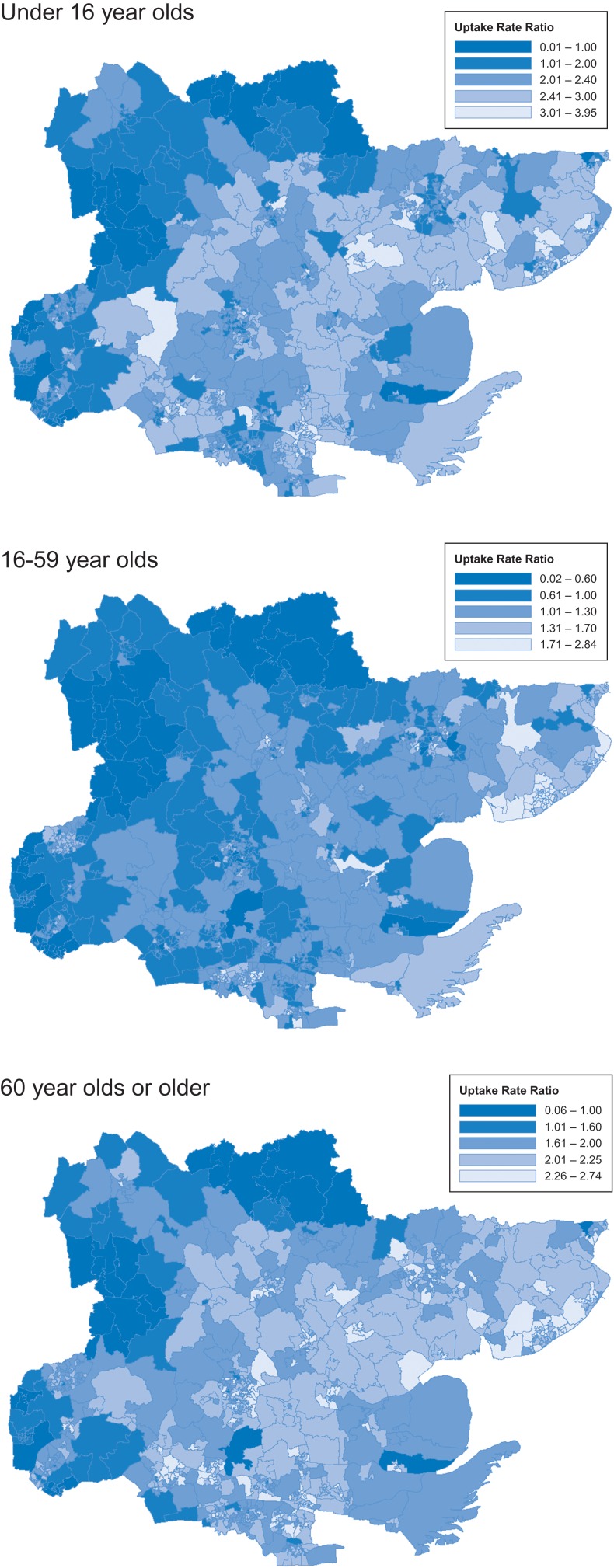
URR in Essex LSOAs in different age groups.

In general, the most deprived LSOAs in Essex tend to be in the coastal areas to the east and to the south of the county along the Thames estuary (Fig. 1). However, due to the differing sizes of LSOAs, as a result of population density, rural LSOAs with larger areas dominate the maps. From the maps, the geographical distribution in uptake of eye examinations for the GOS1 appears to be lower in the north and west of Essex (Fig. 2).

The association between GOS1 uptake and deprivation is better illustrated by looking at the GOS1 uptake ratio by deprivation quintiles (Table 1). The GOS1 uptake rates were higher in the least deprived quintiles of Essex (for the under 16 and 60 and over age groups), although it should be noted that in all quintiles in both the under 16 and 60 and over groups, the observed GOS1 uptake in Essex is higher than would be expected based on the overall England data. For example, the median URR per LSOA in the under 16-year old cohort is 2.29 times higher (IQR 1.98, 2.61) than expected for England overall. In the least derived quintile of under 16-year old in Essex, the median GOS1 URR is 2.49 times higher than expected (IQR 2.21, 2.73) and it is 2.16 times higher than expected (IQR 1.89, 2.44) in the most deprived quintile. In the 16–59-year old age groups, the observed and expected number of tests is broadly similar (Median GOS1 URR (IQR) per LSOA: 1.08 (0.91, 1.31)).

**Table 1:** Unadjusted uptake rates per LSOA by IMD

	No. of LSOAs	Age-group								
		<16			16–59			60 and over		
		Observed no. of tests	Expected no. of tests	Median URR (IQR)	Observed no. of tests	Expected no. of tests	Median URR (IQR)	Observed no. of tests	Expected no. of tests	Median URR (IQR)
Median (IQR) per LSOA: all	872	174 (142, 209)	76.54 (64.23, 92.87)	2.29 (1.98, 2.61)	134.5 (109, 172)	124.73 (110.33, 142.01)	1.08 (0.91, 1.31)	352 (263, 460.5)	179.7 (142.37, 224.46)	2.05 (1.86, 2.18)
Deprivation first quintile (most deprived)	172	190.5 (154, 225.5)	87.92 (72.8, 102.1)	2.16 (1.89, 2.44)	192 (168.5, 229)	129.7 (114.79, 143.09)	1.53 (1.31, 1.79)	276.5 (222, 345.5)	148.66 (121, 177.68)	1.95 (1.79, 2.09)
Deprivation second quintile	170	166 (133, 200)	75.88 (62.89, 89.93)	2.2 (1.95, 2.52)	148 (131, 174)	125.23 (107.01, 141.15)	1.24 (1.05, 1.4)	350 (283, 453)	180.37 (147.54, 226.26)	2 (1.85, 2.15)
Deprivation third quintile	180	162.5 (138, 194)	72.8 (62.49, 87.25)	2.27 (1.95, 2.53)	127 (103, 146.5)	121.85 (109.25, 138.92)	1.04 (0.88, 1.18)	372.5 (294, 463)	198.37 (158.33, 229.63)	2.07 (1.89, 2.17)
Deprivation fourth quintile	179	177 (135, 217)	73.87 (62.09, 93.67)	2.33 (1.95, 2.67)	122 (100, 141)	124.59 (110.33, 144.03)	0.97 (0.82, 1.09)	363 (272, 461)	189.37 (141.69, 229.4)	2.07 (1.83, 2.2)
Deprivation fifth quintile (least deprived)	171	178 (153, 215)	74.14 (62.63, 88.59)	2.49 (2.21, 2.73)	115 (97, 132)	124.15 (108.02, 140)	0.95 (0.83, 1.04)	411 (302, 516)	194.32 (154.29, 233.9)	2.15 (2.04, 2.28)

From the Poisson models, the GOS1 uptake rates were confirmed to be higher in the least deprived quintiles of Essex in both the under 16 and 60 and over age groups compared to the most deprived in the County (Table 2). The GOS1 uptake rate was 26% higher in the least deprived quintile than that seen in the most deprived areas for the under 16-year old (mean Difference in GOS1 URR compared to the most deprived quintile (95% confidence interval (CI)): +0.26 (0.23, 0.30)). Among people aged 60 and over, the GOS1 uptake rates were 15% higher in the least deprived quintile than that seen in the most deprived areas (mean difference in URR compared to most deprived quintile (95% CI): +0.15; 95% CI 0.13, 0.017). For the 16- and 59-year-old group, the GOS1 uptake rate was 62% lower in the least deprived quintile than that seen in the most deprived areas (mean difference in URR compared to most deprived quintile (95% CI): −0.62 (−0.64, −0.60)).

**Table 2:** Mean (95% CI) URRs and differences from most deprived quintile in URRs per LSOA by IMD—Poisson models

Deprivation	Mean URR: URR (95% CI)			Difference in URR (95% CI) from Most deprived quintile		
	<16	16–59	60 and over	<16	16–59	60 and over
First quintile (most deprived)	2.14 (2.11, 2.16)	1.51 (1.5, 1.53)	1.91 (1.9, 1.93)	—	—	—
Second quintile	2.18 (2.15, 2.2)	1.18 (1.17, 1.2)	1.97 (1.96, 1.99)	0.04 (0.01, 0.08)	−0.33 (−0.35, −0.31)	0.06 (0.04, 0.08)
Third quintile	2.14 (2.12, 2.16)	0.98 (0.97, 0.99)	1.92 (1.9, 1.93)	0 (−0.03, 0.04)	−0.53 (−0.56, −0.51)	0 (−0.02, 0.02)
Fourth quintile	2.21 (2.18, 2.23)	0.91 (0.9, 0.92)	1.97 (1.95, 1.98)	0.07 (0.04, 0.1)	−0.6 (−0.62, −0.58)	0.05 (0.03, 0.08)
Fifth quintile (least deprived)	2.4 (2.37, 2.42)	0.89 (0.88, 0.91)	2.07 (2.05, 2.08)	0.26 (0.23, 0.3)	−0.62 (−0.64, −0.6)	0.15 (0.13, 0.17)

There were statistically significant differences in the GOS1 URRs between the 12 Essex local authorities for the three age groups (Table 3), although the differences in URR between the IMD quintiles, in each age-group, remains even on adjustment for differences between Local Councils. On exploring the GOS1 uptake differences by IMD quintile within each of the local authority areas (see Supplementary data, Table), the GOS1 uptake was significantly higher in the least deprived LSOA quintile compared with the most deprived LSOA quintile in each local authority, with the exception of Harlow. However, there were insufficient observations in some quintiles to make similar comparisons in GOS1 URRs in Tendring and Uttlesford local authority areas.

**Table 3:** Mean (95% CI) URRs and differences from most deprived quintile in URRs per LSOA by IMD, adjusted by Local Council—Poisson models

	Mean URR: URR (95% CI)			Difference in URR (95% CI) from most deprived quintile		
	<16	16–59	60 and over	<16	16–59	60 and over
Deprivation						
First quintile (most deprived)	2.03 (2.01, 2.06)	1.41 (1.39, 1.43)	1.79 (1.77, 1.81)	—	—	—
Second quintile	2.11 (2.08, 2.13)	1.13 (1.12, 1.15)	1.89 (1.88, 1.91)	0.07 (0.04, 0.11)	−0.27 (−0.3, −0.25)	0.1 (0.08, 0.12)
Third quintile	2.13 (2.11, 2.15)	0.97 (0.95, 0.98)	1.89 (1.87, 1.9)	0.1 (0.06, 0.13)	−0.44 (−0.46, −0.42)	0.1 (0.07, 0.12)
Fourth quintile	2.26 (2.23, 2.28)	0.93 (0.92, 0.95)	1.99 (1.98, 2.01)	0.23 (0.19, 0.26)	−0.48 (−0.5, −0.45)	0.2 (0.18, 0.23)
Fifth quintile (least deprived)	2.42 (2.39, 2.45)	0.91 (0.9, 0.92)	2.05 (2.04, 2.07)	0.39 (0.35, 0.43)	−0.5 (−0.52, −0.47)	0.26 (0.24, 0.29)
Local council				Difference in URR (95% CI) from Basildon		
Basildon	2.24 (2.21, 2.27)	1.13 (1.11, 1.15)	2.05 (2.03, 2.08)	—	—	—
Braintree	2.16 (2.13, 2.19)	1.09 (1.07, 1.11)	1.85 (1.83, 1.87)	−0.08 (−0.13, −0.03)	−0.04 (−0.06, −0.01)	−0.21 (−0.24, −0.18)
Brentwood	2.31 (2.27, 2.36)	1.03 (1, 1.05)	1.99 (1.96, 2.02)	0.08 (0.02, 0.13)	−0.1 (−0.13, −0.07)	−0.06 (−0.1, −0.02)
Castle Point	2.45 (2.4, 2.49)	1.2 (1.18, 1.23)	2.08 (2.06, 2.11)	0.21 (0.15, 0.26)	0.08 (0.04, 0.11)	0.03 (−0.01, 0.06)
Chelmsford	2.16 (2.13, 2.19)	1.07 (1.05, 1.09)	2.01 (1.99, 2.04)	−0.08 (−0.13, −0.03)	−0.06 (−0.08, −0.03)	−0.04 (−0.07, −0.01)
Colchester	2.31 (2.28, 2.34)	1.04 (1.02, 1.05)	2.05 (2.03, 2.07)	0.07 (0.03, 0.12)	−0.09 (−0.12, −0.07)	0 (−0.03, 0.03)
Epping Forest	1.8 (1.77, 1.84)	0.77 (0.76, 0.79)	1.57 (1.55, 1.59)	−0.44 (−0.48, −0.39)	−0.35 (−0.38, −0.33)	−0.49 (−0.52, −0.46)
Harlow	2.2 (2.15,2.24)	1.05 (1.03, 1.08)	2 (1.97, 2.04)	−0.04 (−0.1, 0.01)	−0.08 (−0.1, −0.05)	−0.05 (−0.09, −0.01)
Maldon	2.32 (2.26, 2.37)	1.2 (1.17, 1.23)	2.08 (2.05, 2.11)	0.08 (0.01, 0.14)	0.07 (0.04, 0.11)	0.02 (−0.02, 0.06)
Rochford	2.61 (2.56, 2.66)	1.17 (1.15, 1.2)	2.07 (2.04, 2.09)	0.37 (0.31, 0.43)	0.05 (0.01, 0.08)	0.01 (−0.02, 0.05)
Tendring	2.7 (2.66, 2.75)	1.44 (1.42, 1.47)	2.2 (2.18, 2.22)	0.47 (0.41,0.52)	0.31 (0.29,0.34)	0.15 (0.12, 0.18)
Uttlesford	1.35 (1.31, 1.38)	0.68 (0.66, 0.7)	1.31 (1.29, 1.33)	−0.89 (−0.94, −0.85)	−0.44 (−0.47, −0.42)	−0.75 (−0.78, −0.71)

## Discussion

### Main findings of this study

There was statistically significant higher uptake of NHS funded sight tests in the least deprived quintile compared with the most deprived quintiles in Essex in the 0–15 and 60 and over cohorts. As would be expected, the uptake of NHS funded sight tests was higher in the most deprived quintile in the working age population, reflecting the fact that receipt of means tested benefits is the most common eligibility GOS criteria recorded in this age-group.^14^

### What is already known on this topic

Associations between socio-economic deprivation and low uptake of sight tests have been observed elsewhere. Van der Pols *et al.*^23^ reported the results of measurement of visual acuity for 1275 people aged 65–101 years, out of the 2059 people recruited for National Diet and Nutrition Survey conducted in 1994–5. About 45% reported having an eye test in the previous year, and 1% reported never having an eye test. Logistic regression analysis showed that respondents of manual social class or lower educational level had less often had an eye test in the previous year compared with higher educated respondents or members of a non-manual household. The logistic regression adjusted for use of spectacles for distance vision as a possible confounding factor. Respondents with a yearly income between £4000 and £6000 were less likely to have had a recent eye test than subjects from a household with an income over £10 000. However, respondents in the lowest household income category (<£4000) had almost the same proportion of having a recent eye test as those in the top two income categories reflecting the fact that when the survey was carried out, only elderly people receiving certain means tested benefits were entitled to an NHS funded sight test.

Given that a sight test is usually the first step in the primary eye care pathway, inequalities in uptake of tests may be associated with inequalities in correction of refractive error. About 9271 eligible members of the 1958 British Birth Cohort^24^ had their visual acuity measured as part of a follow up when they were age 44/45 years. About 37.9% had a previously diagnosed refractive error, but 1.6% of the sample was found to have an undiagnosed refractive error. People with undiagnosed refractive error were more likely to be in a manual rather than a non-manual class, compared to those with a diagnosed refractive error. While this was a strong statistical association, there was no statistically significant trend observed across the social class categories, nor was there a significant association with employment status.

Given that some preventable causes of sight loss are dependent on early detection in the asymptomatic or early symptomatic phase, delayed access of eye examinations can have more significant clinical consequences. In an analysis of data collected as part of the EPIC-Norfolk Eye Study,^25^ low vision was associated with deprivation after adjusting for age, sex, education, social class and cataract surgery, although this effect was mitigated by additionally adjusting for uncorrected refractive error.

Although many people living in the more deprived areas of Essex would be entitled to NHS funded sight tests, widening eligibility may not be a solution. In 2006, all residents of Scotland became eligible for an NHS funded eye examination. Utilizing the British Household Panel Survey, Dickey *et al.*^26^ compared uptake of an NHS funded sight test in the previous 12 months for Scottish respondents to the survey conducted before and after the policy change. Uptake of NHS funded eye examinations was higher among those with a University degree, compared to those with only school qualifications, which in turn was higher than those with no qualifications, both before and after the policy change. However, inequalities in uptake widened after NHS funded eye examinations were available to all Scottish residents. Similarly, there was a positive association between having an eye examination in the previous 12 months and income both before and after the policy change, and these inequalities widened after 2006.

Various studies have attempted to understand barriers to uptake of primary optometric examinations in socio-economically deprived communities: for example in Birmingham,^27^ Leeds,^28,29^ West London,^30^ South Wales, Glasgow, Belfast, Bradford and North East London.^31^ A common finding across all the studies is that the cost of the sight test is not a significant barrier, since most people within these communities had access for free. However, there was a concern about the cost of any optical appliances recommended as a result of the test, and a general mistrust that they would be sold spectacles that they did not need or could not afford. Another common feature was a misunderstanding of the purpose of the eye examination with many people not realizing that it is more than just testing for the need for spectacles and hence that it also have a preventative and screening function for other eye diseases. A glaucoma equity profile conducted in Leeds noted a clear mismatch between the most deprived LSOA and the location of optician's premises.^3^ This reflects the lack of incentives for optometric practices to establish in deprived communities when the business model in the UK requires the cost of eye examinations to be cross subsidized by the sales of optical appliances.^32^

### What this study adds

The analysis of the Essex data adds to the earlier small-area analysis of uptake of NHS funded sight tests in Leeds.^17^ Both analyses demonstrated statistically significant inequalities in uptake of NHS funded sight tests, albeit the reported inequalities in Leeds were greater. However, the Essex data set was significantly larger, covering a bigger and more diverse geographical area, involving more healthcare commissioning localities, over a longer period of time.

### Limitations of this study

The analysis used National GOS1 data for all English regions. As was pointed out above, the data in other English regions are not as complete as for Essex and is derived from only a sample of GOS1 forms. Although NHS Digital do not explain how the samples were selected, there is no reason to expect that this would introduce a selection bias, which would explain the inequalities seen between deprivation quintiles in Essex. Although the uptake in every Essex deprivation quintile is higher than for England overall, we have still identified differences in GOS1 uptake and deprivation.

While the analyses for the Essex local authority areas were similar to that for Leeds, there was been no adjustment for other factors within the Essex data that might explain variation in GOS1 uptake between different areas. While the expected rates account for differences in age between areas and the analysis has been stratified by age, no other factor (e.g. gender, ethnicity, socio-economic circumstances of individuals within those areas) have been accounted for and might explain GOS1 uptake differences, as these data items were not available within the data set.

A further limitation is the exclusions from the data set analysed that might have a potential impact on findings. Those removed as rejected claims (*n* = 795 940, 6.9% removed) and those living outside the region but who had their test within Essex (171 020 (20.0%) removed) were appropriate to exclude for the purpose of the analysis, and so would not potentially alter any associations. However, those 20 584 (2.4%) tests excluded as they did not have a postcode assignable to a LSOA, could be different in terms of deprivation distribution compared with those included in the analysis which might alter any associations. While we cannot compare deprivation distributions we can assess whether there were differences in age distribution. For those included in the analysis the percentages in the three age groups are:  <16—26%, 16–59—21%, 60 and over—53%; compared to 26%, 23% and 51%, respectively, for those 20 584 tests excluded as they did not have a legitimate postcode. Therefore there were proportionally more 16–59-year old and those less 60 and over in those excluded than those subsequently included in the analysis (*χ*^2^ test, *P* < 0.001). However, we cannot ascertain whether these differences in age distribution reflect systematic differences in deprivation distribution and so have an impact on the associations found.

## Conclusions

While improving the quality of secondary care will be important, the key to addressing inequalities in outcomes for preventable sight loss will be to address these barriers to uptake of primary optometric examinations. Further extending eligibility to free eye examinations could increase inequalities not decrease them, as has happened in Scotland. The problem is not likely to be the cost of the sight test, but other barriers such as patient concerns about the cost of spectacles if refractive error is detected. The EPIC-Norfolk Eye Study^25^ concluded that targeting uncorrected refractive error in deprived areas may reduce health inequalities associated with low vision. However, uncorrected refractive error, *per se*, is not a significant public health problem. While uncorrected refractive error is relatively common (1.9% of 48–89 year old in the EPIC-Norfolk Eye Study^33^) and can impact on quality of life, of more concern is severe vision impairment associated with late presentation of wet AMD,^34^ glaucoma,^35^ etc. It will be important to increase public awareness of eye health, especially among deprived communities, and that attending for an eye examination is not just for those with symptoms or who need spectacles. It will also be important to change the business model underpinning optometric practice,^32,36^ so that it becomes economically more viable to provide primary eye care in deprived communities. Directly employing NHS optometrists to work within community settings e.g. within general practice would be another option.^32,36^ The important message is that in order to address inequalities in access and hence inequalities in outcome, public perceptions must be changed so that eye examinations are seen as a ‘healthcare’ rather than a ‘high street experience’.^37^

## Supplementary Material

Supplementary Data
