# Supplementary material for: Geographical inequalities in uptake of NHS funded eye examinations: Poisson modelling of small-area data for Essex, UK
Source: J Public Health (Oxf). 2017 Jun 17;40(2):e171–9. doi: 10.1093/pubmed/fdx058 (PMC6051445; doi:10.1093/pubmed/fdx058)
Supplement: Supplementary Data [file revisedessexgospaperjph28thfebruary2017supplementarydatatable.docx]

**Supplementary data table: Mean (95% Confidence Interval (CI)) Uptake Rate Ratios and Differences from Most Deprived Quintile in Uptake Rate Ratios per Lower Super Output Areas (LSOA) by Index of Multiple Deprivation (IMD) and Local Council - Poisson interaction IMD and Local council models**

|  | **Mean Uptake Rate Ratio: URR (95% CI)** | | | **Difference in URR (95% CI) from Most deprived quintile** | | |
| --- | --- | --- | --- | --- | --- | --- |
|  | **<16** | **16-59** | **60 and over** | **<16** | **16-59** | **60 and over** |
|  |  |  |  |  |  |  |
| Basildon |  |  |  |  |  |  |
| 1st quintile (most deprived) | 2.08 (2.04,2.12) | 1.53 (1.5,1.56) | 1.9 (1.87,1.93) | - | - | - |
| 2nd quintile | 2.15 (2.07,2.24) | 1.2 (1.16,1.25) | 2 (1.94,2.05) | 0.07 (-0.02,0.16) | -0.33 (-0.38,-0.27) | 0.09 (0.03,0.16) |
| 3rd quintile | 2.06 (1.98,2.15) | 1.04 (1,1.08) | 2.09 (2.03,2.15) | -0.02 (-0.11,0.07) | -0.49 (-0.54,-0.44) | 0.19 (0.12,0.25) |
| 4th quintile | 2.39 (2.25,2.53) | 0.95 (0.88,1.01) | 2.02 (1.95,2.1) | 0.31 (0.16,0.45) | -0.58 (-0.65,-0.51) | 0.12 (0.04,0.2) |
| 5th quintile (least deprived) | 2.53 (2.46,2.6) | 0.93 (0.9,0.97) | 2.22 (2.18,2.27) | 0.45 (0.37,0.53) | -0.6 (-0.64,-0.55) | 0.32 (0.27,0.38) |
| Braintree |  |  |  |  |  |  |
| 1st quintile (most deprived) | 2.18 (2.09,2.28) | 1.55 (1.48,1.62) | 2.08 (2,2.16) | - | - | - |
| 2nd quintile | 2.06 (1.99,2.12) | 1.17 (1.13,1.21) | 1.87 (1.83,1.91) | -0.13 (-0.24,-0.01) | -0.38 (-0.46,-0.31) | -0.21 (-0.3,-0.12) |
| 3rd quintile | 1.97 (1.91,2.04) | 0.92 (0.89,0.96) | 1.74 (1.7,1.77) | -0.21 (-0.32,-0.1) | -0.63 (-0.71,-0.55) | -0.34 (-0.43,-0.26) |
| 4th quintile | 2.2 (2.14,2.27) | 0.93 (0.9,0.97) | 1.8 (1.76,1.84) | 0.02 (-0.1,0.13) | -0.62 (-0.7,-0.54) | -0.28 (-0.37,-0.19) |
| 5th quintile (least deprived) | 2.6 (2.5,2.7) | 1.1 (1.05,1.16) | 2.08 (2.01,2.15) | 0.41 (0.27,0.55) | -0.45 (-0.54,-0.36) | 0 (-0.1,0.11) |
| Brentwood |  |  |  |  |  |  |
| 1st quintile (most deprived) | 2.12 (1.95,2.29) | 1.2 (1.09,1.31) | 1.94 (1.8,2.07) | - | - | - |
| 2nd quintile | 2.37 (2.22,2.52) | 1.2 (1.12,1.28) | 1.77 (1.69,1.86) | 0.25 (0.02,0.48) | 0 (-0.14,0.14) | -0.17 (-0.32,-0.01) |
| 3rd quintile | 2.19 (2.09,2.29) | 0.96 (0.91,1.01) | 1.93 (1.86,1.99) | 0.07 (-0.13,0.27) | -0.24 (-0.36,-0.12) | -0.01 (-0.16,0.14) |
| 4th quintile | 2.26 (2.14,2.39) | 0.79 (0.73,0.84) | 1.96 (1.89,2.03) | 0.14 (-0.07,0.35) | -0.42 (-0.54,-0.3) | 0.02 (-0.13,0.17) |
| 5th quintile (least deprived) | 2.61 (2.53,2.7) | 0.93 (0.89,0.97) | 2.22 (2.18,2.27) | 0.49 (0.3,0.68) | -0.27 (-0.38,-0.16) | 0.28 (0.14,0.43) |
| Castle Point |  |  |  |  |  |  |
| 1st quintile (most deprived) | 2.22 (2.09,2.34) | 1.74 (1.65,1.83) | 1.92 (1.84,2) | - | - | - |
| 2nd quintile | 2.22 (2.13,2.31) | 1.27 (1.22,1.32) | 2.04 (1.99,2.09) | 0 (-0.15,0.16) | -0.47 (-0.57,-0.36) | 0.11 (0.02,0.21) |
| 3rd quintile | 2.5 (2.41,2.6) | 1.07 (1.03,1.12) | 2.09 (2.04,2.14) | 0.29 (0.13,0.44) | -0.66 (-0.77,-0.56) | 0.16 (0.07,0.26) |
| 4th quintile | 2.7 (2.58,2.81) | 1.13 (1.07,1.19) | 2.15 (2.09,2.2) | 0.48 (0.31,0.65) | -0.61 (-0.71,-0.5) | 0.23 (0.13,0.32) |
| 5th quintile (least deprived) | 2.54 (2.4,2.68) | 0.91 (0.85,0.98) | 2.19 (2.12,2.26) | 0.32 (0.14,0.51) | -0.82 (-0.93,-0.71) | 0.26 (0.16,0.37) |
|  |  |  |  |  |  |  |
|  | **Mean Uptake Rate Ratio: URR (95% CI)** | | | **Difference in URR (95% CI) from Most deprived quintile** | | |
|  | **<16** | **16-59** | **60 and over** | **<16** | **16-59** | **60 and over** |
| Chelmsford |  |  |  |  |  |  |
| 1st quintile (most deprived) | 1.96 (1.84,2.08) | 1.41 (1.32,1.5) | 1.85 (1.74,1.95) | - | - | - |
| 2nd quintile | 1.96 (1.85,2.06) | 1.1 (1.04,1.16) | 2.04 (1.97,2.11) | 0 (-0.16,0.16) | -0.31 (-0.41,-0.21) | 0.2 (0.07,0.32) |
| 3rd quintile | 2.21 (2.14,2.28) | 1.01 (0.98,1.05) | 1.92 (1.88,1.96) | 0.25 (0.11,0.39) | -0.4 (-0.49,-0.3) | 0.08 (-0.03,0.19) |
| 4th quintile | 2.21 (2.15,2.27) | 0.88 (0.85,0.91) | 2.1 (2.06,2.15) | 0.26 (0.12,0.39) | -0.53 (-0.62,-0.44) | 0.26 (0.15,0.37) |
| 5th quintile (least deprived) | 2.38 (2.32,2.43) | 0.97 (0.95,1) | 2.17 (2.14,2.2) | 0.42 (0.29,0.55) | -0.44 (-0.53,-0.35) | 0.32 (0.22,0.43) |
| Colchester |  |  |  |  |  |  |
| 1st quintile (most deprived) | 2.1 (2.02,2.17) | 1.31 (1.27,1.36) | 1.89 (1.83,1.96) | - | - | - |
| 2nd quintile | 2.29 (2.23,2.35) | 1.1 (1.07,1.14) | 2.05 (2.01,2.1) | 0.19 (0.1,0.29) | -0.21 (-0.27,-0.15) | 0.16 (0.08,0.23) |
| 3rd quintile | 2.22 (2.14,2.3) | 1.05 (1,1.09) | 2.02 (1.98,2.07) | 0.13 (0.02,0.23) | -0.27 (-0.33,-0.21) | 0.13 (0.05,0.21) |
| 4th quintile | 2.34 (2.29,2.4) | 0.94 (0.91,0.97) | 2.15 (2.11,2.18) | 0.25 (0.16,0.34) | -0.37 (-0.42,-0.32) | 0.25 (0.18,0.32) |
| 5th quintile (least deprived) | 2.71 (2.59,2.82) | 0.82 (0.78,0.86) | 2.11 (2.05,2.16) | 0.61 (0.48,0.75) | -0.49 (-0.55,-0.43) | 0.21 (0.13,0.29) |
| Epping Forest |  |  |  |  |  |  |
| 1st quintile (most deprived) | 1.53 (1.45,1.61) | 0.85 (0.81,0.9) | 1.32 (1.27,1.38) | - | - | - |
| 2nd quintile | 1.93 (1.86,2) | 0.94 (0.9,0.98) | 1.58 (1.53,1.62) | 0.4 (0.3,0.5) | 0.09 (0.03,0.15) | 0.25 (0.18,0.32) |
| 3rd quintile | 1.76 (1.7,1.82) | 0.67 (0.64,0.7) | 1.5 (1.46,1.53) | 0.23 (0.13,0.33) | -0.18 (-0.23,-0.12) | 0.17 (0.11,0.24) |
| 4th quintile | 1.7 (1.62,1.79) | 0.69 (0.65,0.73) | 1.66 (1.61,1.71) | 0.17 (0.06,0.29) | -0.16 (-0.23,-0.1) | 0.34 (0.26,0.41) |
| 5th quintile (least deprived) | 1.97 (1.88,2.06) | 0.73 (0.68,0.77) | 1.76 (1.71,1.81) | 0.44 (0.32,0.56) | -0.13 (-0.19,-0.06) | 0.44 (0.36,0.51) |
| Harlow |  |  |  |  |  |  |
| 1st quintile (most deprived) | 2.08 (2.03,2.13) | 1.4 (1.37,1.44) | 1.87 (1.83,1.91) | - | - | - |
| 2nd quintile | 2.12 (2.03,2.21) | 1.14 (1.09,1.19) | 1.96 (1.9,2.02) | 0.04 (-0.07,0.14) | -0.26 (-0.33,-0.2) | 0.09 (0.01,0.16) |
| 3rd quintile | 2.08 (1.93,2.24) | 0.98 (0.9,1.06) | 2.07 (1.97,2.18) | 0 (-0.16,0.16) | -0.43 (-0.51,-0.34) | 0.2 (0.09,0.31) |
| 4th quintile | 2.25 (2.09,2.41) | 0.93 (0.84,1.02) | 1.92 (1.77,2.07) | 0.17 (0,0.33) | -0.47 (-0.57,-0.37) | 0.05 (-0.11,0.2) |
| 5th quintile (least deprived) | 1.93 (1.72,2.13) | 0.83 (0.7,0.95) | 1.88 (1.62,2.15) | -0.16 (-0.37,0.06) | -0.58 (-0.71,-0.45) | 0.01 (-0.25,0.27) |

|  | **Mean Uptake Rate Ratio: URR (95% CI)** | | | **Difference in URR (95% CI) from Most deprived quintile** | | |
| --- | --- | --- | --- | --- | --- | --- |
|  | **<16** | **16-59** | **60 and over** | **<16** | **16-59** | **60 and over** |
| Maldon |  |  |  |  |  |  |
| 1st quintile (most deprived) | 2.19 (2.02,2.37) | 1.65 (1.53,1.78) | 1.9 (1.79,2) | - | - | - |
| 2nd quintile | 2.1 (1.98,2.22) | 1.13 (1.06,1.19) | 1.85 (1.79,1.92) | -0.09 (-0.3,0.12) | -0.52 (-0.67,-0.38) | -0.04 (-0.17,0.08) |
| 3rd quintile | 2.35 (2.25,2.44) | 1.12 (1.07,1.17) | 2.1 (2.05,2.15) | 0.15 (-0.05,0.35) | -0.53 (-0.66,-0.39) | 0.21 (0.09,0.33) |
| 4th quintile | 2.41 (2.29,2.54) | 1.1 (1.03,1.16) | 2.21 (2.14,2.29) | 0.22 (0,0.43) | -0.55 (-0.7,-0.41) | 0.32 (0.19,0.45) |
| 5th quintile (least deprived) | 2.48 (2.33,2.64) | 1.12 (1.04,1.2) | 2.27 (2.18,2.36) | 0.29 (0.06,0.52) | -0.53 (-0.68,-0.38) | 0.37 (0.23,0.51) |
| Rochford |  |  |  |  |  |  |
| 1st quintile (most deprived) | 1.83 (1.57,2.09) | 1.49 (1.28,1.7) | 1.93 (1.66,2.21) | - | - | - |
| 2nd quintile | 2.24 (2.11,2.36) | 1.22 (1.15,1.29) | 1.98 (1.91,2.04) | 0.41 (0.12,0.69) | -0.28 (-0.5,-0.05) | 0.04 (-0.24,0.33) |
| 3rd quintile | 2.78 (2.62,2.94) | 1.22 (1.14,1.3) | 2.17 (2.09,2.26) | 0.95 (0.64,1.25) | -0.27 (-0.5,-0.04) | 0.24 (-0.05,0.53) |
| 4th quintile | 2.74 (2.65,2.83) | 1.06 (1.02,1.11) | 2.1 (2.05,2.15) | 0.91 (0.64,1.18) | -0.43 (-0.64,-0.21) | 0.16 (-0.12,0.45) |
| 5th quintile (least deprived) | 2.94 (2.86,3.03) | 0.97 (0.93,1.01) | 2.23 (2.18,2.27) | 1.11 (0.84,1.38) | -0.52 (-0.74,-0.31) | 0.29 (0.01,0.57) |
| Tendring |  |  |  |  |  |  |
| 1st quintile (most deprived) | 2.54 (2.48,2.6) | 1.95 (1.91,2) | 2.05 (2.02,2.08) | - | - | - |
| 2nd quintile | 2.64 (2.57,2.72) | 1.56 (1.52,1.61) | 2.2 (2.16,2.23) | 0.1 (0,0.2) | -0.39 (-0.45,-0.33) | 0.15 (0.1,0.19) |
| 3rd quintile | 2.68 (2.59,2.78) | 1.29 (1.24,1.34) | 2.15 (2.11,2.19) | 0.14 (0.03,0.25) | -0.67 (-0.73,-0.6) | 0.1 (0.05,0.16) |
| 4th quintile | 2.41 (2.27,2.55) | 1.14 (1.07,1.22) | 2.24 (2.17,2.31) | -0.13 (-0.28,0.02) | -0.81 (-0.89,-0.73) | 0.19 (0.12,0.27) |
| 5th quintile (least deprived) | * | * | * | * | * | * |
| Uttlesford |  |  |  |  |  |  |
| 1st quintile (most deprived) | * | * | * | - | - | - |
| 2nd quintile | 0.93 (0.78,1.07) | 0.62 (0.52,0.71) | 1.14 (1.01,1.27) | * | * | * |
| 3rd quintile | 1.23 (1.15,1.31) | 0.55 (0.51,0.6) | 1.38 (1.31,1.44) | * | * | * |
| 4th quintile | 1.58 (1.52,1.63) | 0.71 (0.68,0.74) | 1.52 (1.48,1.55) | * | * | * |
| 5th quintile (least deprived) | 1.27 (1.21,1.33) | 0.47 (0.44,0.5) | 1.1 (1.07,1.14) | * | * | * |
|  |  |  |  |  |  |  |

* No estimates as no observations in these quintiles for Tendring (5^th^ quintile) or Uttlesford (1^st^ quintile), with the later resulting in no comparison with the most deprived quintile and other quintiles in Uttlesford.
